# Consecutive record-breaking high temperatures marked the handover from hiatus to accelerated warming

**DOI:** 10.1038/srep43735

**Published:** 2017-03-03

**Authors:** Jingzhi Su, Renhe Zhang, Huijun Wang

**Affiliations:** 1 State Key Laboratory of Severe Weather, Chinese Academy of Meteorological Sciences, Beijing, 100081, China; 2 Institute of Atmospheric Sciences, Fudan University, Shanghai, 200433, China; 3 Collaborative Innovation Center on Forecast and Evaluation of Meteorological Disasters, Nanjing University for Information Science and Technology, Nanjing, 210044, China

## Abstract

Closely following the hiatus warming period, two astonishing high temperature records reached in 2014 and 2015 consecutively. To investigate the occurrence features of record-breaking high temperatures in recent years, a new index focusing the frequency of the top 10 high annual mean temperatures was defined in this study. Analyses based on this index shown that record-breaking high temperatures occurred over most regions of the globe with a salient increasing trend after 1960 s, even during the so-called hiatus period. Overlapped on the ongoing background warming trend and the interdecadal climate variabilities, the El Niño events, particularly the strong ones, can make a significant contribution to the occurrence of high temperatures on interannual timescale. High temperatures associated with El Niño events mainly occurred during the winter annual period. As the Pacific Decadal Oscillation (PDO) struggled back to its positive phase since 2014, the global warming returned back to a new accelerated warming period, marked by the record-breaking high temperatures in 2014. Intensified by the super strong El Niño, successive high records occurred in 2015 and 2016. Higher frequencies of record high temperatures would occur in the near future because the PDO tends to maintain a continuously positive phase.

The warming tendency of the global mean surface temperature (GMST) slowed after 1999 relative to the trend observed for the 60-year period from 1951 to 2012, thus indicating a hiatus of global warming^1^,^2^,^3^. The reasons for the supposed hiatus on human-caused warming include natural forcing (volcanic and solar) and internal variability^4^,^5^. The steady background warming trend could sometime be obscured by large natural variabilities^6^. Many features clearly show that global warming continued during the hiatus period. The GMST reached new record highs in 2014 and 2015^7^,^8^. High recorded temperatures were still observed in a number of regions around the globe after 2000, and temperature and precipitation extremes increased over land regions in recent decades^9^,^10^, reflecting an apparent anthropologic contribution to climate change.

The 10 highest recorded GMSTs until 2015 occurred in 2015, 2014, 2010, 1998, 2009, 2005, 2013, 2002, 2006, and 2003 (Fig. 1), and seven of these years are in the so-called hiatus period (2000–2013), thereby reflecting a steady warming background trend. Although the GMST warming rate decreased from 0.14 °C/decade over the period of 1979–1998 to 0.11 °C/decade over the period of 1999–2012, the GMST tendency was still positive over the last decade, which indicated that global warming continued in recent decades. Moreover, certain facts related to the hiatus are disputed, including the possible artifacts of data biases^3^. In this paper, another aspect of the continued warming is investigated to provide a new perspective.

## Observed Results

**Figure 1:**
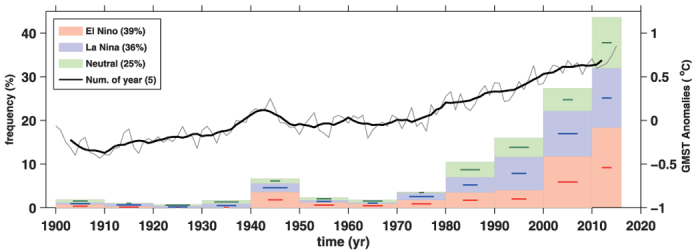
Relative frequency of top 10 record (NT10) of annual mean temperatures for each decade. The NT10 is divided into three subgroups (El Niño, La Niña, and neutral years) as indicated by the legend. The NT10 frequency in the 2010 s is weighted by 10/6, as only the six years of 2010–2015 are used in the calculation. The number of El Niño, La Niña, and neutral events during a decade is indicated by the thick line in each bar. The annual mean GMST is plotted as a thin black line, and its seven-year moving average is plotted as a thick black line.

To highlight the high temperature events, the number of times that an event of top 10 high record (NT10) of annual mean temperature events is recorded for each grid location, and the NT10 frequency for each decade are investigated (see Methods). The annual NT10 changes of global mean temperature are consistent with the GMST time series (Supplementary Fig. S1), and the global area-weighted NT10 frequency shows a steady increase after the 1950 s on a decadal time scale (Fig. 1).

Following the rapid increase of GMSTs from 1970–1997, the NT10 of global temperatures also increased. The NT10 frequency was 11% and 16% in the 1980 s and 1990 s respectively, which represents accelerated warming decades. Conversely, the NT10 frequency was approximately 2% to 4% during the 1950 s, 1960 s and 1970 s, a period that has also been designated as a global warming hiatus. After a warming acceleration period in the first half of the 20^th^ century (1920 s–1930 s), the GMST reached a relative maximum during the first half of the 1940 s (Supplementary Fig. S1), with the NT10 frequency reaching 7% in the 1940 s.

Although the GMST warming tendency weakened after 2000, the frequency of high record temperatures increased during the hiatus period, with the NT10 frequency increasing to 27% in the 2000 s. Moreover, the NT10 frequency accumulated (see Methods) in the short period 2000–2015 (53%) constituted a large proportion of the total NT10 frequency (100%) of temperatures recorded over the entire period (1900–2015), with the NT10 frequency reaching 26% in the six years from 2010 to 2015. Unless a significant cooling period occurs prior to 2020, then the NT10 frequency of global temperatures for the 2010 s will be approximately 43%.

**Figure 2:**
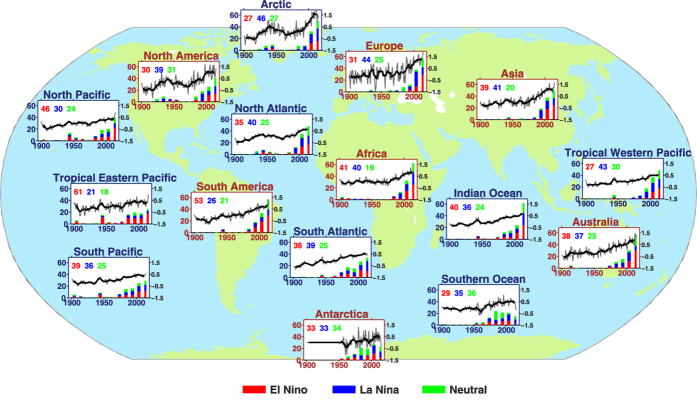
Same as in Fig. 1 but for each continent and ocean. The NT10 percentages in El Niño, La Niña, and neutral years are indicated by red, blue, and green numbers, respectively, in each panel. This figure is generated using Matlab (R2010) and the mapping package M_Map (Byrne, D., A mapping package for Matlab, 2014, https://www.eoas.ubc.ca/~rich/map.html).

A significantly non-uniform spatial distribution occurred in the decadal changes of NT10 frequency (Fig. 2). In most areas, the top 10 temperatures mainly occurred after the 1950 s and the accumulated NT10 frequency of global temperatures over the first half of the 20^th^ century was less than 11%. High temperature records could still be observed in particular regions in the first half of the 20^th^ century. Relatively high accumulated NT10 frequencies were observed from the 1900 s to 1940 s in the Arctic (19%), North America (19%) and the North Atlantic (14%), thus reflecting the accelerated warming period in the first half of the 20^th^ century.

The NT10 frequency generally increased in most areas of the globe after 2000. The accumulated NT10 frequency from 2000 to 2015 reached a high level (above 50%) in all seven continents except Antarctica: Europe (72%), Africa (70%), Asia (69%), South America (62%), Australia (61%), and North America (51%). Compared with the NT10 frequencies on continents, relatively lower accumulated NT10 frequencies were observed in the oceans, with high values in the Arctic (65%), the tropical western Pacific (65%), the North Atlantic (64%), and the Indian Ocean (61%). The NT10 frequencies on continents is generally higher than that in oceans, which is consistent with the explanation that the warming hiatus from 1999 to 2012 was primarily caused by cooling in the oceans, particularly the tropical eastern Pacific^4^. For almost all continents and oceans, the accumulated NT10 frequency was commonly higher from 2000 to 2015 than from 1960 to 1999. However, the accumulated NT10 frequency in the tropical eastern Pacific decreased from 41% over the period 1960–1999 to 33% over the period 2000–2015, which was primarily caused by the lower sea surface temperature anomalies (SSTAs) in this region in recent decades because of the La Niña-like pattern of negative Pacific Decadal Oscillation (PDO)/Interdecadal Pacific Oscillations. Influenced by the negative PDO, a relatively lower accumulated NT10 frequency was also observed in the North Pacific (44%) and the South Pacific (43%) after 2000.

As demonstrated by the steadily increasing NT10 frequency of global temperatures for each decade after the 1960 s (Fig. 1), the NT10 frequency was higher during the 2000 s than in any of the prior decades for almost all of the continents and oceans with one exception: a notable reduction of NT10 frequency in the Southern Ocean after the 1980 s. The NT10 frequency in the Southern Ocean increased to a maximum of 24% during the 1980 s, decreased gradually during the 1990 s (21.4%) and 2000 s (20.9%), and declined to only 9% from 2010 to 2015, which was markedly lower compared with that of the global temperature (43%) during the same period. In the areas adjacent to the Southern Ocean, lower NT10 frequencies were also observed from 2010 to 2015 in the South Pacific (14%), the South Atlantic (20%), and Antarctica (14%) compared with the values recorded in these regions in the 2000 s.

On an interannual timescale, GMST variability can be induced by the El Niño-Southern Oscillation (ENSO). The percentage of top 10 high record GMSTs that occurred in El Niño years was approximately 39% for the entire period, while these values were lower in La Niña years (36%) and neutral years (25%) (Fig. 1). Because an El Niño/La Niña event usually peaks in the boreal winter, the winter annual mean is used here instead of the commonly-used calendar annual mean (See methods). The winter annual mean is defined as the mean of the values recorded for the period from early June to the late May, which covers the entire period of El Niño/La Niña events from development to decay (Supplementary Information). The percentage of NT10 winter annual mean temperatures was 44% for El Niño years, 32% for La Niña years and 24% for neutral years (Supplementary Fig. S2).

The influence of El Niño events on NT10 frequencies also shows clear spatial variations (Fig. 2; Supplementary Fig. S3). Because positive SSTAs are mainly located in the tropical eastern Pacific during an El Niño event, a high NT10 (63%) for the winter annual mean temperature occurred in the El Niño years in the tropical eastern Pacific. The ENSO can influence the global temperature via atmospheric teleconnection bridges, and can cause climate anomaly in the mid-latitude Pacific/North American regions and in the global tropical regions through generating Walker circulation changes^11^,^12^. A high NT10 for the winter annual mean temperature in El Niño years can also be found in South America (61%), the Indian Ocean (49%), Australia (53%), and Africa (55%), which corresponds to the ENSO teleconnection pattern (Supplementary Fig. S4). Based on the SSTAs spatial pattern, the El Niño events can be classified into two or three types: the eastern-Pacific (EP) type, the central-Pacific (CP) type, and the mixed type^13^, and each type El Niño may lead different atmosphere circulation changes^14^. As a result, some differences of NT10 distribution can be found between during different type El Niño. In EP-type El Niños, high NT10 located in the eastern equatorial Pacific and the mid-latitude Pacific/North America regions. In CP-type El Niños, relatively lower NT10 distribution can be found in the central equatorial Pacific, and the NT10 values also reduced in the mil-latitude region (Supplementary Fig. S5).

Indeed, the NT10 frequency in El Niño years was reduced from a high value in previous decades to a lower value after 2010 in both the Indian Ocean and the Arctic. The NT10 frequencies in the Indian Ocean in La Niña and neutral years over the period from 2010 to 2015 was relatively larger than that over the period from 2000 to 2009, thus reflecting the prominent warming trend in the Indian Ocean in recent decades^15^,^16^,^17^. Such more NT10 frequencies in Indian ocean may be resulted by the asymmetry and skewness in ENSO forcing^17^. On the other hand, the Indian Ocean SSTs may also have a damping effect on the ENSO variability and cycle^18^. Hence, the interaction between the Indian and Pacific oceans should be considered to investigate the reason of the Indian ocean warming. The NT10 frequency in El Niño years also reduced after 2010 in the Arctic region, reflecting the phenomenon of Arctic warming amplification^19^. The occurrence of high temperature during El Niño events in the Arctic and the Indian Ocean reduced after 2010, exactly revealing that the tremendous effect of the undergoing global warming. As a result, the contribution of the steady warming trend on high temperature records gradually overwhelmed the contribution of the El Niño, at least in the regions of the Arctic and the Indian Ocean. The mean annual surface temperature in the Indian Ocean showed an increasing trend of 0.08 °C/decade during the period of 1979–1998 and 0.20 °C/decade during the period of 1999–2015. As a close neighbour to the Indian ocean, Australia has also been undergoing a high warming trend (0.41 °C/decade) during the period 1999–2015. Such a drastic warming trend during the period from 1999 to 2015 was also observed in the Arctic (0.51 °C/decade).

Several processes are thought to contribute to Arctic amplification, including local radiative effects from increased greenhouse-gas forcing^20^,^21^, changes in the snow- and ice-albedo feedback induced by a diminishing cryosphere^22^. The increasing anthropogenic greenhouse gases were supposed to be the primary cause for the salient warming in the Indian ocean since 1970 s^23^,^24^. The cross-equatorial cell in the Indian ocean weakened after 1950 s, which favored that warming by reducing the southward (northward) transport of warm surface water from (cold thermocline water into) the north Indian Ocean^25^. The increased odds of Australian summer extremes were investigated with much attention. It was supposed that human contribution was substantial for the local extreme warming in the Australia, while natural climate variations alone, including ENSO, are unlikely to explain the record temperature^26^.

Even under the overwhelming global warming background, some exception can also be found in the regions near Antarctica. Paused warming trend can be found in the Southern Ocean (−0.08 °C/decade), the South Pacific (0.00 °C/decade), and Antarctica (0.08 °C/decade). Slow warming in the Southern Ocean under the global warming background has been attributed to a large oceanic thermal inertia, with very deep surface mixed layer over there^27^. Furthermore, the Southern Ocean warming may be delayed by the equatorward heat transport in upper oceanic layer^28^, or be caused by negative SSTAs in the tropical Pacific associated with negative PDO phase after 2000^29^.

## Explanation for the Increasing Record-Breaking High Temperatures

Definitely, the undergoing global warming provides the crucial driving force for the increasing occurrence of the record-breaking high temperatures. The background warming trend can be modulated by natural climate variabilities on interannual-to-decadal timescales. In the hiatus period after 2000, the warming speed slowed down, mainly influenced by the negative PDO. However, the NT10 frequency showed a significant increasing from lower values during the previous “accelerated warming” decades (11% in 1980 s and 16% in 1990 s; Fig. 1) to a higher value during the “hiatus” decades (27% in 2000 s). From this aspect, the new defined index NT10 here has its own advantage to effectively reveal the dominant warming trend. On the other hand, the GMST may cause some confusion by itself, just as the well-known hiatus term was defined based on the GMST.

Considering the steady increasing NT10 in each decade from 1960 s to 2000 s under the global warming background, it should not be surprising to see a higher NT10 in the 2010 s. However, the NT10 frequency already showed an amazing increment, compared with previous decades, only in the first six years of the 2010 s (Fig. 1), which was mostly contributed by the high NT10 in the years of 2014 and 2015 (Figure S1). Besides the primary contribution of continuous warming trend, the new record high temperatures in 2014 and 2015 might be also favored by the strong 2014/2015 El Niño events. Particularly, the spatial distribution of winter annual mean temperatures in 2015 (Supplementary Fig. S6) shown an ENSO teleconnection pattern in the eastern tropical Pacific and regions near North America.

However, it is apparent that the high temperatures in 2014/2015 cannot be explained only by the strong El Niño events. Although the interannual-to-interdecadal variabilities of the detrended GMSTs cannot be totally linearly explained by the ENSO and the PDO (Supplementary Fig. S7), the regress estimated results match the detrended GMSTs one interannual variabilities quite well. Significant larger residual terms can be found in the 2014/2015 than that in 1997. A comparison between the two super strong El Niño events in 2015 and 1997 can explain how much contribution to the high GMSTs in 2015 actually made by the El Niño. The winter annual mean temperatures in 1997/1998 ranked as a top 5 high record, and the spatial distributions both of the NT10 and the temperature anomalies in 1997/1998 had a resemblance to the ENSO teleconnection pattern (Supplementary Figs S6c and S4d). On the other hand, the NT10 winter annual mean temperatures in 2015/2016 displayed a striking difference. Salient warming can be found in the Indian ocean, Eurasian continent, and the Arctic regions, all of which were absent in the 1997/1998 pattern. Such differences between 2015 and 1997 clearly demonstrated that the record-breaking high temperatures in 2015 were not determined only by the super El Niño event, and also implied that the global warming has been accelerated in the last two-three years.

The above analyses state that the undergoing global warming background is the first and primary driving force for the record-breaking high temperatures in recent years. However, it is desirable to investigate the mechanisms of the ENSO’s influence on the record high temperatures, particularly the two record-breaking high temperatures in 2014 and 2015.

The investigation starts from the comparison of the influences of different type El Niño event. Although significant global warming can be found associated with the EP El Niño events, the CP type and the mixed type El Niño could not induce such global warming signals^30^. Since the observed EP El Niño events tended to have a stronger amplitude than those of CP-type or mixed-type El Niño events, three strongest El Niño events (1982, 1997, and 2015) are chosen to represent the classical EP-type El Niño.

**Figure 3:**
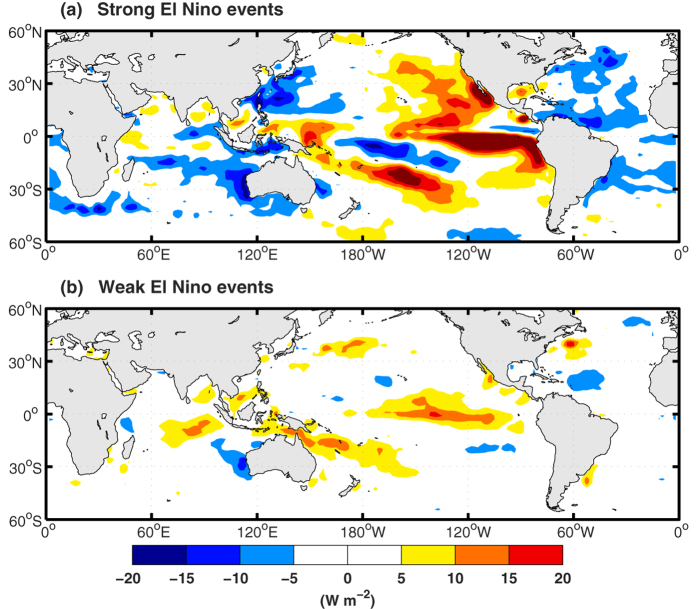
The composited latent heat fluxes during of (**a**) strong (1982, 1997, 2015) El Niño events and (**b**) moderate (1991, 1994, 2002, 2004, 2006) El Niño events. The heat fluxes are composited based on the dataset from NCEP reanalysis 1, NCEP reanalysis 2, ERA-interim, and OAFlux. This figure is generated using Matlab (R2010) and the mapping package M_Map (Byrne, D., A mapping package for Matlab, 2014, https://www.eoas.ubc.ca/~rich/map.html).

During El Niño events, the latent heat fluxes are the dominant term among the surface heat fluxes changes in the tropical Pacific region (not shown). Heat comes out of the Pacific Ocean mainly in the form of moisture that is evaporated and which subsequently warms the atmosphere when rains out, releasing the latent energy^31^. Significant differences can be found in the latent heat fluxes pattern between the strong/EP El Niño events and the CP-type events (Fig. 3). Associated with the strong SSTAs during EP El Niño, large latent heat fluxes were released from the ocean into the atmosphere in the eastern equatorial Pacific. Significant latent heat fluxes released by the ocean also located in the eastern subtropical North and South Pacific, which may be related poleward propagations as oceanic Kelvin waves of the equatorial warm waters and then their westward propagations as Rossby waves^32^. On the other hand, the latent heat fluxes anomalies are much weaker during weak/CP El Niño events, and those weak anomalies are only confined to the central-eastern equatorial Pacific. Such differences about the latent heat fluxes demonstrated that the atmosphere tends to be warmed with a greater amplitude during/after EP-type El Niño events than that of CP-type El Niño events, as much more moisture is evaporated into the atmosphere during EP-type El Niño events.

Furthermore, there are some differences associated with the record-breaking high temperatures in 2014 and 2015. Significant latent heat fluxes were released by the ocean to the atmosphere in 2015 (Supplementary Fig. S8), a typical pattern following the strong/EP El Niño cases. On the other hand, the latent heat fluxes released by ocean in 2014 were much weaker than those in 2015. In fact, the high record in 2014 was related to the extreme warming in the northeastern Pacific and the accumulated warming in the western Pacific (Supplementary Fig. S6b), an effective sign for the PDO phase transition from negative phase to positive phase.

**Figure 4:**
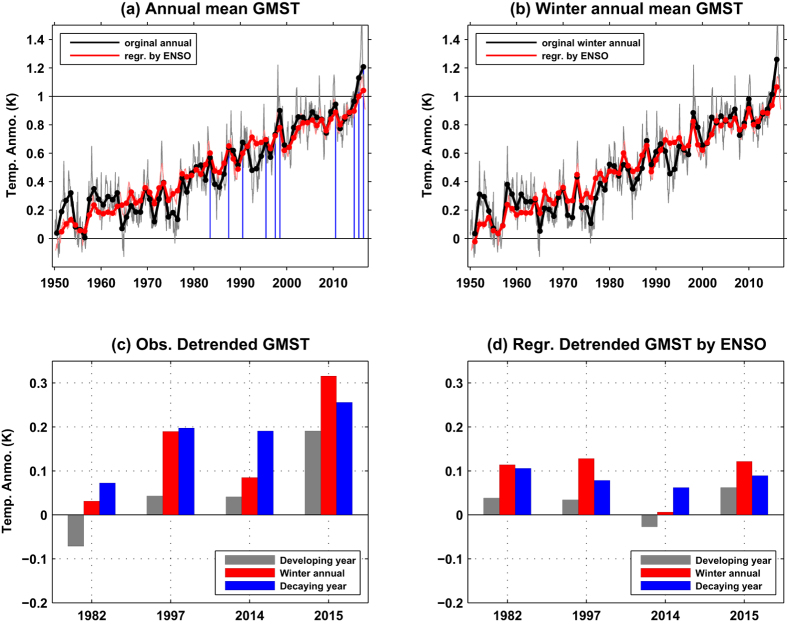
(**a**) The observed (black) annual mean GMST and the estimated values (red) based a linear regression against the temperature anomalies averaged on the region NINO3 (5°N–5°S, 150°W–90°W). (**b**) Same as (**a**) but for the winter annual mean values. (**c**) The observed detrended GMST averaged during the developing year (gray), the winter annual mean (red), and averaged during the decaying year (blue) for each El Niño events. (**d**) Same as (**c**) but for the estimated values based a linear regression against the temperature anomalies in NINO3. Those years with record-breaking high GMST are indicated by blue thin bars in (**a**). The anomalies are calculated based on the average over the period of 1880–1899, representing the pre-industrial level.

The contribution of El Niño on record-breaking high temperatures deserves a much deeper investigation. First, an estimate for the monthly temperature anomalies on each grid was calculated based on regress analysis by the temperature averaged in the NINO3 region, with a lag time of 3 months. Then the GMST can be estimated as the global area-weighted mean of the regress estimated values on each grid (see Methods; Fig. 4a,b). The regress estimate results match the observed GMST well, with significant interannual increments associated with strong/EP El Niño events. It should be noted that the moderate El Niños also apply some effects on the global temperature variabilities in certain regions (Supplementary Fig. S5b), while such weak contributions are hard to be detected when using the GMST index. To focus on the influence by strong El Niño events on the interannual variabilities of GMST, the annual mean values for 1982, 1997, 2014 and 2015 are shown in Fig. 4c,d. As the temperature anomalies in NINO regions tend to cause high GMST with about 3-month lag time (Supplementary Fig. S9d), apparent influence of El Niño events on GMST can be observed in their decaying years. The observed detrended GMST reach 0.07 °C, 0.20 °C, and 0.26 °C in 1983, 1998, and 2016 (blue bars in Fig. 4c), and the estimated contribution by El Niño is about 0.17 °C, 0.14 °C, and 0.09 °C, respectively (blue bars in Fig. 4d). Hence, approximately one-half of the interannual variability of detrended GMST associated with strong El Niño events could be explained by the El Niño. During the developing year of those El Niño events, the El Niños still make some contribution (gray bars in Fig. 4d) to the weak interannual variabilities of detrended GMST (gray bars in Fig. 4c). When evaluated by the index of winter annual mean, the observed detrended GMST variabilities (0.03 °C in 1982/1983, 0.19 °C in 1997/1998, 0.32 °C in 2015/2016; red bars in Fig. 4c) can also be largely explained by El Niño (0.14 °C, 0.17 °C, and 0.13 °C respectively; red bars in Fig. 4d).

For the recent record-breaking high temperatures in 2014, the direct contribution from El Niño is very weak (−0.01 °C), which matches the above results based on the estimation with the latent heat fluxes changes. For the case in 2015, it is estimated that the El Niño could lead anomalous global warming of 0.06 °C, which is only about one third of the observed result (0.19 °C). Furthermore, the huge increment (0.15 °C) of GMST from 2014 to 2015 cannot be explained only by the El Niño (0.07 °C). Those results reaffirm that the recent record-breaking high temperature in 2014 and 2015 marked a new accelerated warming after the prior hiatus period. As the background warming trend (about 0.13 °C/decade) has been removed, the astonishing record-breaking high temperatures during 2014–2015 can then be regarded as the combination of the contribution by the interdecadal variabilities and the El Niños. As the El Niño cannot explain the high GMST increment in 2014 and only explain one third of the GMST increment in 2015, the residual terms then can be treated as the contribution by interdecadal variabilities, which can be mostly attributed to the positive PDO.

The background warming trend is about 0.13 °C/decade. Driven by this ongoing warming trend, more new high records would occur in the future even without any strong El Niño events. Hence, the record-breaking high temperature, with a magnitude of that in 2014/2015/2016, would definitely occur sooner or later. This is simple and is a fact. Hence, we think it is not appropriate to estimate the El Niño’s contribution to the total (original) global warming (only about 10% by El Niño for the high record in 2015 if evaluated by this scheme). Instead, the estimate of the El Niño’s contribution for global warming was given based on the detrended GMST in this study. By this way, the El Niño may apply more than one third contribution to the huge increment of GMST in 2015 on interannual timescale, and also apply important contribution to the high GMST in 2016.

In short, the increasing frequency of record-breaking high temperatures in recent years cannot simply be explained only by the El Niño events. There are three key points related with high temperatures: (1) the overwhelming warming trend; (2) the decadal variability; (3) the interannual variability. The 2014 high record warming can be treated as the starting of one new “accelerated warming period” (warming trend overlapped by the positive PDO). The 2015/2016 high record warming can be treated as the extreme hot event during an accelerated warming period aroused by a super El Niño.

## Discussion

As the overwhelming global warming background has been playing a major driving force for the temperature trend, it is reasonable to deduce that more and more record-breaking high temperatures would occur, as a “new normal” state^26^, in the next several decades. However, some fluctuations, overlapped on the robust warming trend, would still be expected for the occurrence frequency of record high temperatures. The exact time of appearance and the exact spatial location of the future high temperatures would be influenced by the natural climate variabilities on interannual-to-interdecadal timescales, e.g., the ENSO and the PDO. Particularly, super strong El Niños can cause huge increment (more than 0.10 °C in 1983, 1998, 2016) of GMST on interannual timescales, which would dim the background warming trend (about 0.13 °C/decade) for about one decade. For example, if calculated based on the period of 1983–1993 (after the 1982 strong El Niño), a fictitious “paused” warming trend (0.09 °C/decade) can also be derived, comparable to that during 1999–2012. The so-called hiatus period after super strong El Niño in 1997/1998 would not be so much striking if there were no the negative-phase PDO just occurring during the same period. In another word, the huge contribution of strong El Niño on interannual timescale could led a fictitious feature of “paused” warming on decadal timescales. And, such fictitious “paused” warming can be concealed or be highlighted by decadal climate variabilities depending on their positive or negative phase. Anyway, the record high local temperatures over the globe have never stopped to occur during the hiatus warming period, which is demonstrated by the increasing NT10 with an apparent amplitude. From this aspect, the NT10 index seems to be more qualified to monitor the warming status than the GMST itself.

**Figure 5:**
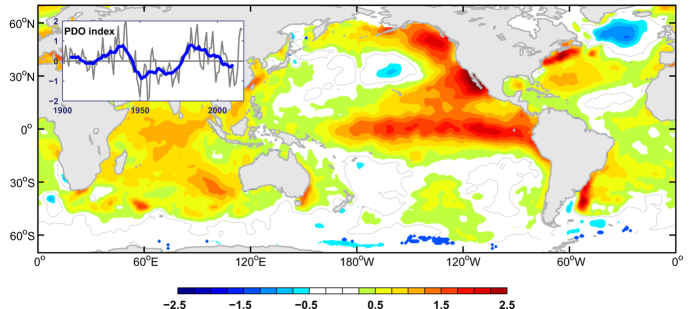
Annual mean sea surface temperature during 2015. The Pacific Decadal Oscillation index is plotted as a thin gray line, and its 11-year moving average is plotted as a thick blue line. This figure is generated using Matlab (R2010) and the mapping package M_Map (Byrne, D., A mapping package for Matlab, 2014, https://www.eoas.ubc.ca/~rich/map.html).

Three record-breaking high temperatures have come into being in 2014, 2015, and 2016 consecutively. Such unprecedented “triple jump” of record-breaking high GMST was quite unique since record-keeping began in 1880, and the GMST has exceeded the pre-industrial conditions by more than 1 °C already^33^ (Fig. 4a). Such high record temperatures were associated with the extraordinary warming in the northeastern Pacific^34^ (Fig. 5), which helped the PDO switch to a positive phase in the recent two years (Fig. 5). Previous studies showed that the warming hiatus from 1999 to 2012 was mainly caused by the negative PDO pattern^4^. The PDO or the Interdecadal Pacific Oscillation pattern seems to remain in a positive phase for several years, which indicates that global warming has been shifting from a hiatus phase to an acceleration phase since about 2014. Based on an initialized decadal prediction model, it was predicted that the PDO has been transiting from the previous negative phase to positive phase after 2013^35^. Furthermore, there are no any effective indications to show when would the local warming amplification in the Arctic and Indian Ocean regions be terminated in the near future. And the temporarily delayed warming in the Southern Ocean would finally change into a warming state comparable to that in the Northern Hemisphere^36^. Thus, additional high record temperatures could occur in the near future. More and more new record-breaking high temperatures are expected to occur in the following several decades.

## Methods

### Observational data sets

GISTEMP data products were used for the calculations^37^. The global mean surface temperature was calculated using grid points for which GISTEMP data were available for the entire period. To calculate the annual mean sea surface temperature anomalies in Fig. 5, HadISST data^38^ were used. The anomalies are calculated relative to 1961–1990 baseline. The Pacific Decadal Oscillation index was obtained from the JISAO, University of Washington. The heat fluxes are combined based on the dataset from NCEP reanalysis 1, NCEP reanalysis 2, ERA-interim, and OAFlux.

### Frequency calculation

For each grid (*i* = 1, *Nx*; *j* = 1, *Ny, with an area of A*_*i*,*j*_), the annual mean temperatures and the corresponding years are sorted in ascending order. Then, the years that correspond to the top 10 recorded temperatures were obtained. To represent the occurrence of high temperatures, the number of times that an event of a top 10 record (NT10) of annual mean temperature occurred in certain period (one decade in this study;*t* = 1900–1909, …, 2010–2019) was counted as the absolute frequency [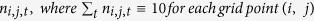)]. The relative frequency of the occurrence of top 10 record temperatures was similarly calculated for each decade 
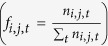
. Then, the NT10 frequency for a certain region [one continent or ocean basin in this study; *R*_*s*_, which includes many grids))] is defined as the area-weighted mean relative frequency for all of the grids in that region [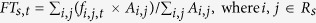)]. Note that the sum of the NT10 frequencies for certain region is 100% for the entire period (1900–2015). Because the last decade only contains 6 years until 2015, the NT10 frequency shown in the figures is weighted by 10/6; therefore, the bar area is proportional to the frequency at different time intervals. Furthermore, three subgroups (El Niño, La Niña and normal years) of NT10 frequencies are calculated based on the corresponding years of the top 10 recorded annual mean temperatures.

### Accumulated frequency definition

To investigate the NT10 frequency for a period (e.g., two continuous decades), the sum of the decadal NT10 frequency for that period is defined as the accumulated frequency.

### ENSO definition

ENSO events since 1950 are defined by the December-January-February (DJF) averaged sea surface temperature anomalies (SSTAs) in the NINO3.4 region (5°N–5°S, 120°W–170°W) obtained from NCEP/CPC. An El Niño (La Niña) event is defined if the DJF averaged NINO 3.4 SSTAs exceeding 0.5 °C (drops below −0.5 °C). For period of 1900–1949, ENSO events are defined by the October-November-December-January-February (ONDJF) averaged SSTAs in the NINO 3.4 region. An El Niño (La Niña) event is defined if the ONDJF averaged SSTAs exceeding 0.5 °C (drops below −0.5 °C). Due to the high number of SSTAs in the NINO 3.4 region in winter of 2014/2015, the 2014/2015 event was defined as an El Niño.

#### Regress estimate for GMST

For each grid (*i* = 1, *Nx*; *j* = 1, *Ny, with an area of A*_*i*,*j*_), an estimate (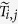) for the monthly surface temperature anomalies is calculated based on the regress analysis of the temperature values against the temperature anomalies averaged in NINO3 (5°N–5°S, 150°W–90°W). Then an estimated GMST can be obtained as 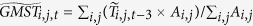)], with a leading time of 3 months for NINO3. All the fields are linearly detrended. To be shown in Fig. 4a,b, the linear trend is overlapped. Similar results can be obtained when calculated based on NINO3.4 region.

## Additional Information

**How to cite this article**: Su, J. *et al*. Consecutive record-breaking high temperatures marked the handover from hiatus to accelerated warming. *Sci. Rep.* **7**, 43735; doi: 10.1038/srep43735 (2017).

**Publisher's note:** Springer Nature remains neutral with regard to jurisdictional claims in published maps and institutional affiliations.

## Supplementary Material

Supplementary Information
