# Supplementary material for: Consecutive record-breaking high temperatures marked the handover from hiatus to accelerated warming
Source: Sci Rep. 2017 Mar 3;7:43735. doi: 10.1038/srep43735 (PMC5335651; doi:10.1038/srep43735)
Supplement: Supplementary Information [file srep43735-s1.pdf]

Supplementary Information for

Consecutive record-breaking high temperatures marked the handover from hiatus to accelerated warming

Jingzhi Su*, Renhe Zhang, Huijun Wang
correspondence to: sujz@camscma.cn

This file includes:

Supplementary Text
Figs. S1-S9

Supplementary Text

Relationships among ENSO, high record temperature, and winter-annual mean

On an interannual timescale, the GMST variability can be induced by the El Niño-Southern Oscillation. The super El Niño of 1997/1998 was followed by a record high GMST in 1998. The occurrence of high recorded temperatures is generally related to El Niño events. Note that El Niño events tend to mature during the boreal winter season. High recorded annual mean temperatures may occur in the year before an El Niño peak phase, whereas others may occur later in the year after an El Niño peak phase, which is usually a La Niña developing year. The top 5 high recorded annual mean temperatures occurred in 2015, 2014, 2010, 1998, and 2005, and 1998, 2010, and 2005 were La Niña developing years. In fact, the high temperatures in 1998, 2010, and 2005 were mainly caused by the relative warming in the first half of the year, which was influenced by the prior El Niño events during their peak-decaying

phase. During the latter half of 1998, 2010, and 2005, the SSTAs were replaced by a La Niña pattern. Consequently, the GMST in the second half of the year was lower than that in the first half of the year for El Niño-decaying-La Niña-developing years.

To describe the influence of El Niño events, particularly their peaks, on the GMST, an alternative to the ordinary method of calculating the annual mean is provided here. The winter annual mean is used instead of the commonly used annual mean. The winter annual mean is defined as the mean during the period from early June to late May, which covers the entire period of El Niño events from their development phase to decay phase.

These methods facilitate the detection of the influence of El Niño on the high temperature record. The top 5 high record winter annual mean GMSTs occurred in 2015/16, 2014/15, 2009/10, 2006/07, 2013/14, and 1997/98. Hence, the high temperature records in several La Niña developing years can be easily explained. For example, the recorded high GMST in 1998 was mainly caused by higher temperatures during the first half of the year, which corresponded to the decaying phase of the 1997/1998 El Niño. The Niño 3.4 index dropped below -0.5°C after July 1998, thus forming a La Niña event.

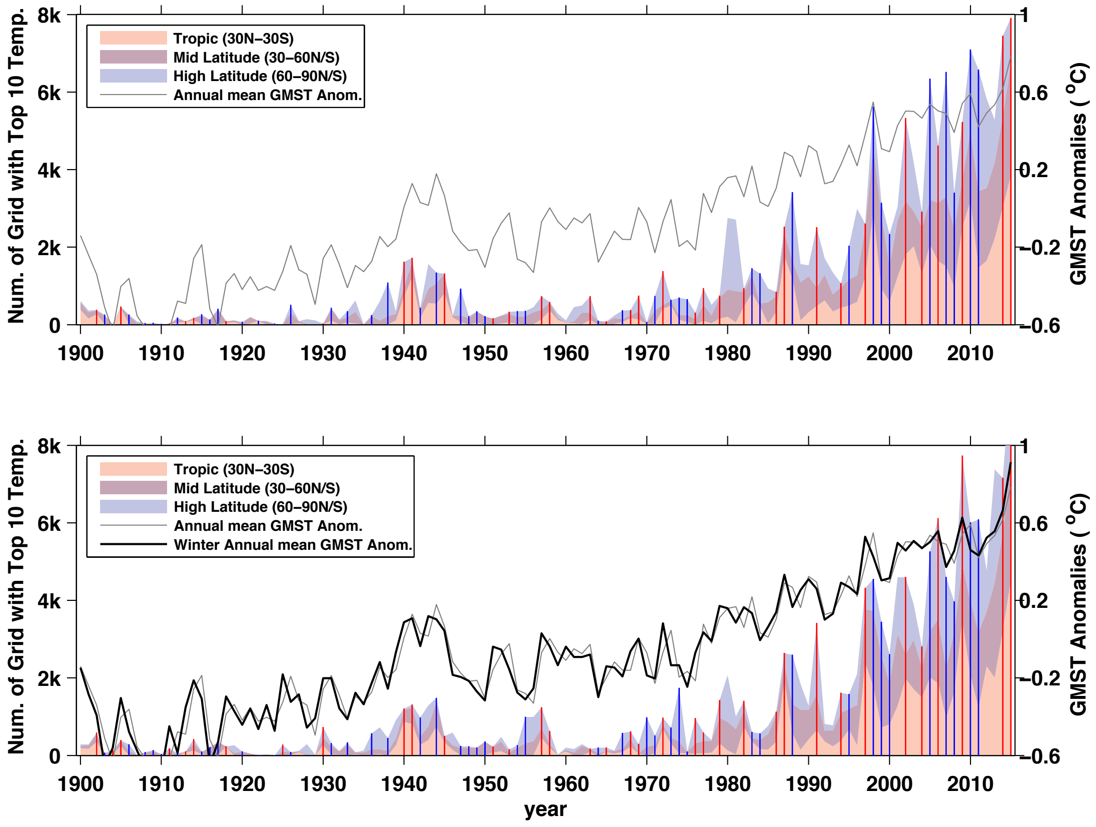

Figure S1. Number of grids with top 10 record (upper) annual mean temperatures and (bottom) winter annual mean temperatures. The global area-weighted annual mean temperature (winter annual mean temperature) is plotted as a thin gray (thick black) line. The winter annual mean is defined as the mean during the period from early June to late May. The vertical thin red (blue) lines indicate the El Niño (La Niña) years.

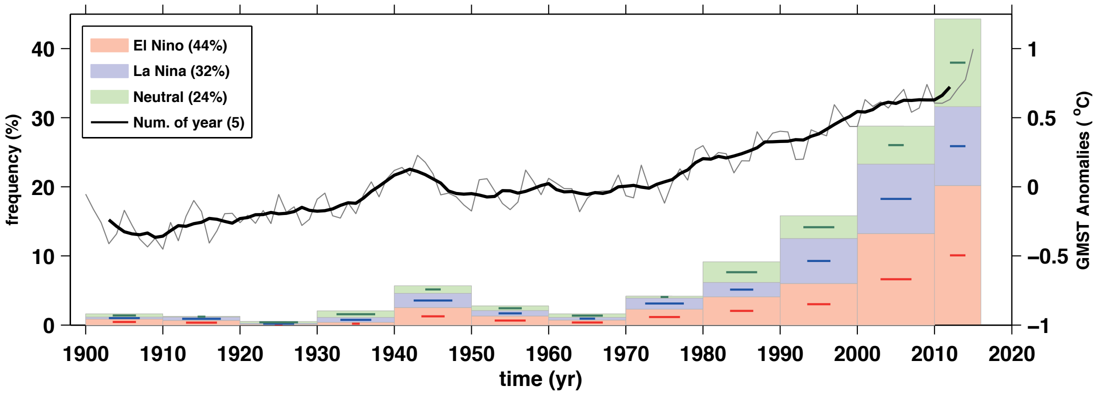

Figure S2. Same as in Figure 1 but for the winter annual mean temperatures. The winter annual mean is defined as the mean during the period from early June to late May.

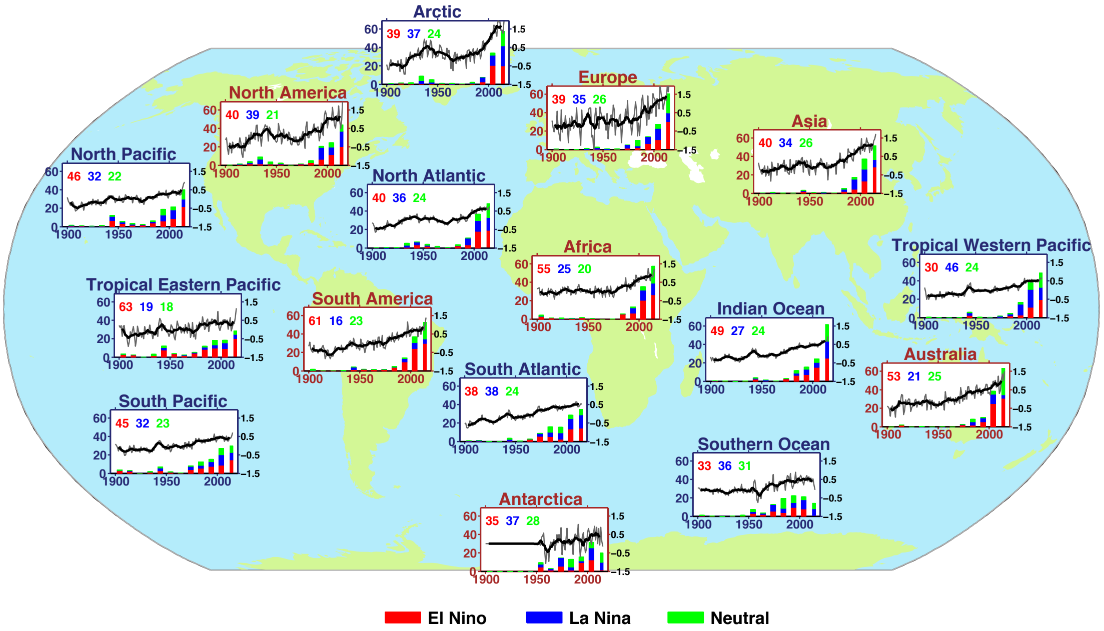

Figure S3. Same as in Figure 2 but for the winter annual mean temperatures. The winter annual mean is defined as the mean during the period from early June to late May. This figure is generated using Matlab (R2010) and the mapping package M_Map (Byrne, D., A mapping package for Matlab, 2014, https://www.eoas.ubc.ca/~rich/map.html).

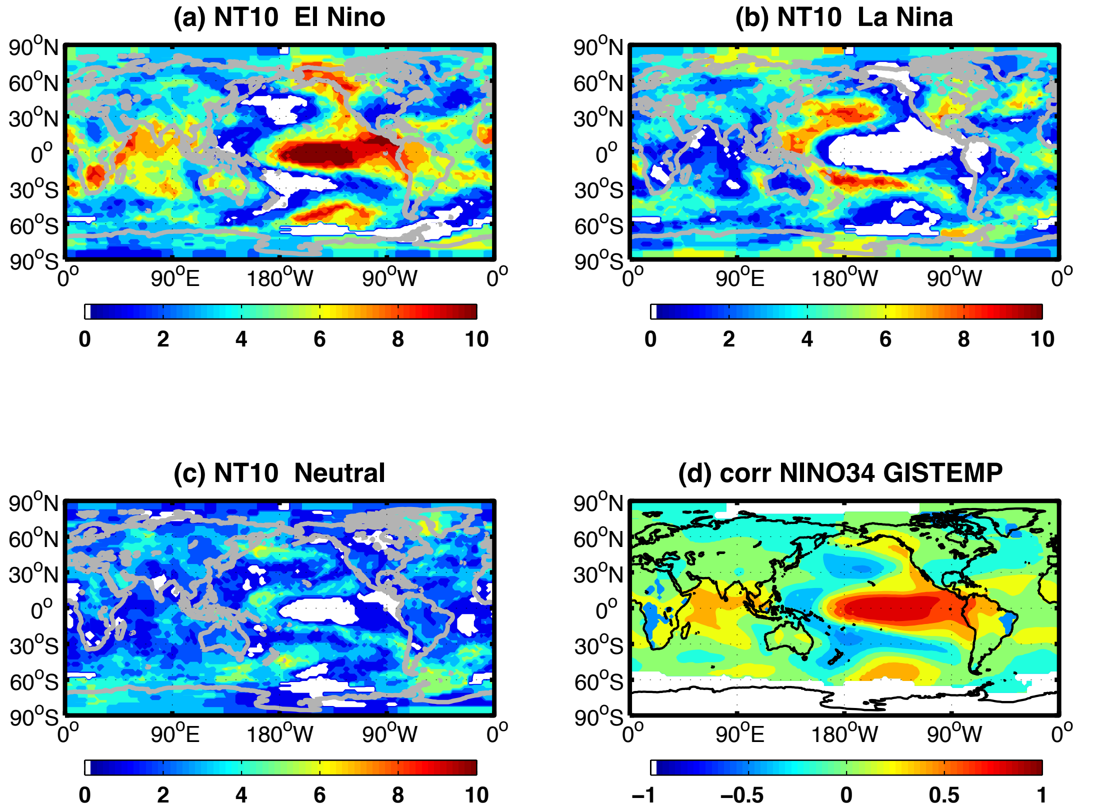

Figure S4. The NT10 spatial distribution of the winter annual mean temperatures in (a) El Niño years, (b) La Niña years, and (c) neutral years. The correlation of monthly surface temperature with NINO3.4 index is plotted in (d). The figures are generated using Matlab (R2010) and the mapping package M_Map (Byrne, D., A mapping package for Matlab, 2014, https://www.eoas.ubc.ca/~rich/map.html).

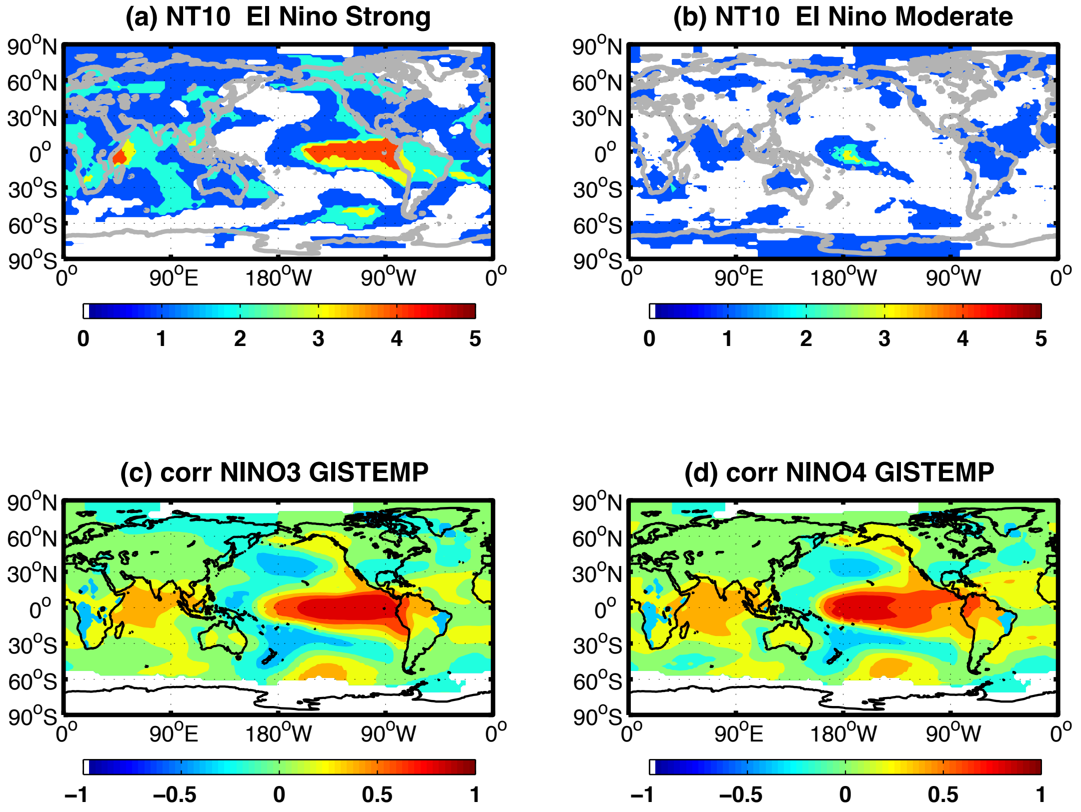

Figure S5. Same as Figure S4, but for composites of (a) strong (1982, 1997, 2015) El Niño events and (b) moderate (1991, 1994, 2002, 2004, 2006) El Niño events. The values in (b) is weighted by 3/5, as the number of years for composites is 3 (5) for strong (moderate) El Niño. The figures are generated using Matlab (R2010) and the mapping package M_Map (Byrne, D., A mapping package for Matlab, 2014, https://www.eoas.ubc.ca/~rich/map.html).

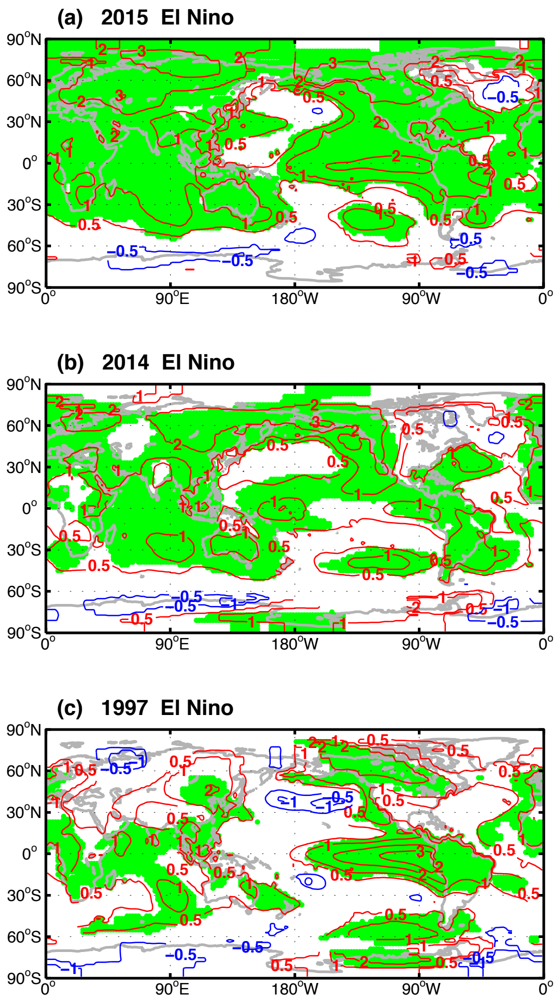

Figure S6. The anomalies (contour lines) of the winter annual mean temperatures in (a) 2015 El Niño, (b) 2014 El Niño, and (c) 1997 El Niño. The green dots indicate where the local temperature ranked in the top 10 high records. The figures are generated using Matlab (R2010) and the mapping package M_Map (Byrne, D., A mapping package for Matlab, 2014, https://www.eoas.ubc.ca/~rich/map.html).

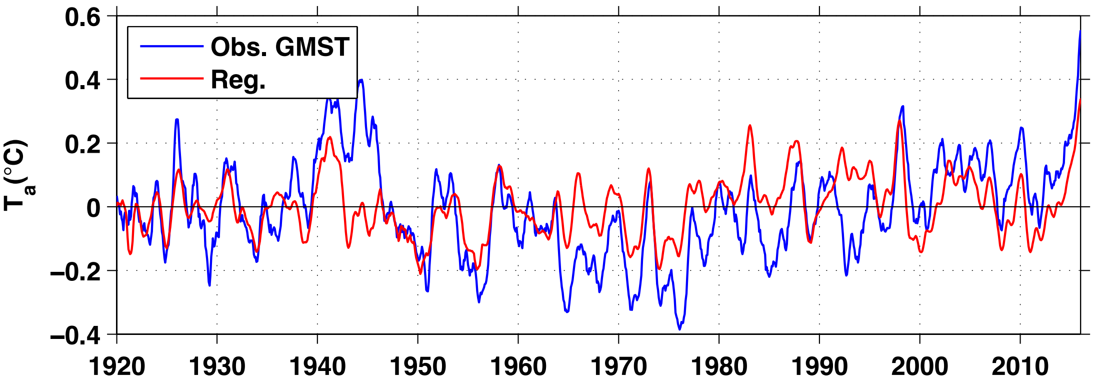

Figure S7. The observed GMST (blue line) and the regressed values (red line) based on 2-month-leading NINO3.4 index and PDO index. Before regress analysis, the GMST time series are linearly detrended. The NINO3.4 (PDO) index time series are smoothed with 7-month (3-year) moving average.

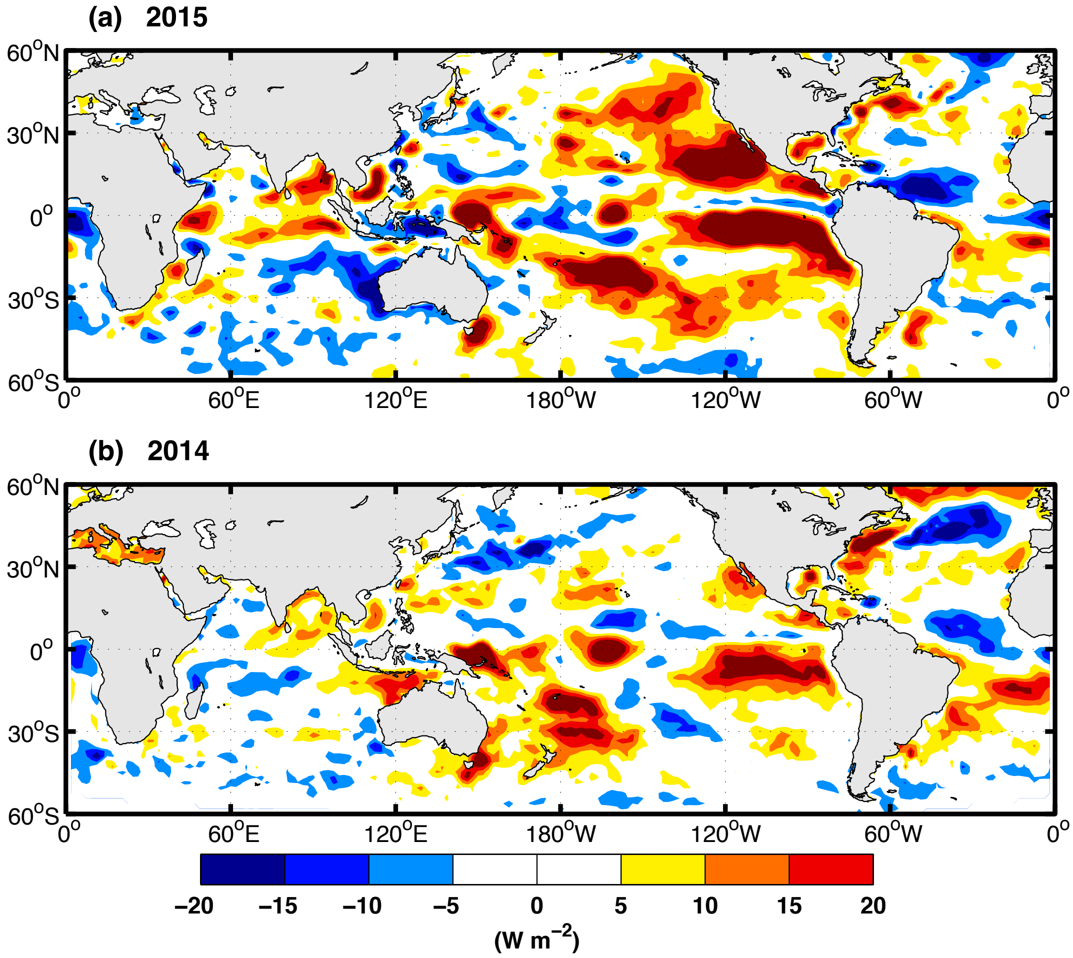

Figure S8. Same as Figure 3, but for the (a) 2015 year and (b) 2014 year.

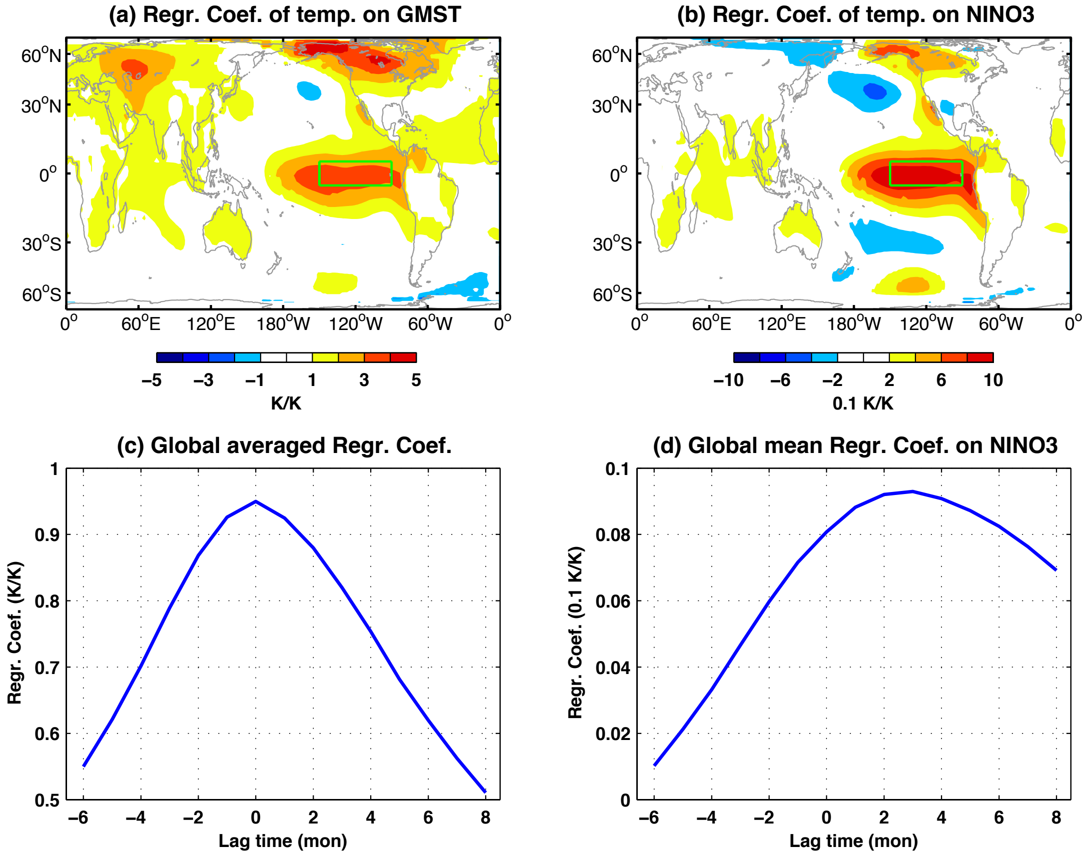

Figure S9. (a) The regress coefficient of monthly surface temperature anomalies on GMST. (b) The regress coefficient of monthly surface temperature anomalies on the temerature anomalies averaged in NINO3 (5°N–5°S, 150°W–90°W) with a leading time of 3 months. (c) The global area-weighted mean regress coefficient of surface temperature anomalies on GMST as a function of lead and lag, and positive values mean GMST leading. (d) Same as (c) but for values regressed on temperature anomalies averaged in NINO3. The analyses are based on the GISTEMP during the period of 1950-2015. All the values are linearly detrended before regression calculation. The NINO3 region is indicated by green line.
